# Impact of ischemic lesion on sleep related connectivity in the sensorimotor cortex

**DOI:** 10.3389/fnins.2025.1661458

**Published:** 2025-11-04

**Authors:** Maria Giovanna Canu, Federico Barban, Michela Chiappalone, Gabriele Arnulfo, Vinícius Rosa Cota

**Affiliations:** ^1^Department of Informatics, Bioengineering, Robotics and Systems Engineering, Università degli Studi di Genova, Genova, Italy; ^2^IRCCS Ospedale Policlinico San Martino, Genova, Italy; ^3^Rehab Technologies Lab, Istituto Italiano di Tecnologia, Genova, Italy; ^4^Child Neuropsychiatry Unit, IRCCS Istituto Giannina Gaslini, Full Member of the ERN EpiCARE, Genova, Italy; ^5^Department of Electronic Engineering, Maynooth University, Maynooth, Ireland

**Keywords:** local field potential, phase locking value, phase amplitude coupling, connectivity, neuroplasticity, slow-wave sleep

## Abstract

Ischemic events can cause cell death and tissue loss, leading to the impairment of neural circuitry by disconnection of its neural substrates. However, the highly plastic properties of the nervous system can provide recovery by boosting circuital redundancies or triggering functional adaptation/repurposing of closely related networks. In this context, understanding how ischemic brain lesions reorganize circuits directly or indirectly connected to the injury site is crucial for developing therapeutic approaches, particularly neuroprostheses based on neurostimulation for brain-rewiring. Furthermore, it is also fundamental to consider the sleep–wake cycle in such an inquiry, considering its well-established role as bearer of key mechanisms of neuroplasticity. This study aimed to investigate how an ischemic lesion in the rat’s primary motor cortex affects the connectivity of areas involved in the sensorimotor loop, specifically the premotor cortex (RFA) and the primary somatosensory cortex (S1), during sleep. We analyzed Local Field Potentials recorded during slow-wave sleep in rats with and without ischemic lesions. Functional connectivity and cross-frequency interactions were quantified using Phase Locking Value (PLV) and Phase-Amplitude Coupling (PAC) analyses, respectively. Our findings revealed a marked increase in PAC 7 days after the lesion, followed by a partial return toward baseline levels at 14 days post-lesion. These results suggest a transient reorganization of network dynamics associated with early recovery processes. The observed changes provide insights into spontaneous post-stroke plasticity during sleep and identify potential electrophysiological biomarkers of recovery. Our findings may contribute to the design of sleep-integrated neurostimulation strategies to promote motor rehabilitation after stroke.

## 1 Introduction

Stroke is the second leading cause of death and the third leading cause of disability worldwide, with ischemic strokes representing nearly 87% of all stroke cases (Novakovic et al., 2009; Benjamin et al., 2017; Saini et al., 2021; Feigin, 2022). According to the World Health Organization, approximately 13 million people suffer a stroke annually, and of these, about 5 million are left permanently disabled (Tursunov, 2017; Lindsay et al., 2019). In addition to the acute loss of function due to brain damage, stroke triggers a cascade of neurobiological alterations which extend beyond the lesion site, compromising network-level interactions critical for coordinated brain functions (Ovadia-Caro et al., 2013; Dijkhuizen et al., 2014; Alia et al., 2021). As a result, the behavioral consequences of stroke reflect both of local and widespread activity changes, leading to sensorimotor or cognitive impairments often described as disconnection syndromes (Siegel et al., 2016; Salvalaggio et al., 2020; Averna et al., 2022). In this context, functional connectivity is increasingly recognized as an indicator of such impairments and a potential biomarker for recovery (Wu et al., 2015; Cassidy et al., 2022).

Among the physiological processes involved in post-stroke recovery, sleep plays a pivotal role. Indeed, recent evidence has highlighted the intricate bidirectional relationship between stroke and sleep. On one hand, good sleep quality promotes neuroplasticity and support recovery processes following stroke (Facchin et al., 2020; Kim et al., 2022); on the other, stroke often leads to multifaceted disturbances in sleep architecture, including both short term aberrant increases of slow oscillations, as well as reduced slow-wave sleep and fragmented sleep patterns in the long term, among others (Hermann and Bassetti, 2003; Kim et al., 2022; Sharma et al., 2022). Given that sleep is a major modulator of synaptic remodeling and memory consolidation (Abel et al., 2013; Mikutta et al., 2019; Puentes-Mestril et al., 2019), these alterations may negatively affect the restoration of neural networks. Despite this evidence, the mechanistic relationship between post-stroke sleep dynamics and the reorganization of cortical networks remains largely unexplored, particularly with respect to frequency-dependent interactions among spared regions.

Neuroplasticity, the brain’s ability to reorganize its structure and function in response to injury, plays a fundamental role in post-stroke recovery (Zotey et al., 2023; Singh, 2024). Ischemic events trigger a series of neuroinflammatory processes that are correlated to neuroplastic changes (Kriz and Lalancette-Hébert, 2009; Sandvig et al., 2018; Alsbrook et al., 2023). Furthermore, neuroplasticity is tightly linked to sleep, as many of the mechanisms underlying synaptic strengthening, pruning, and circuit reshaping occur preferentially during specific sleep stages (Klinzing et al., 2019). Disruptions of these dynamics following stroke may therefore hinder the re-establishment of functional connectivity.

In this study, we used rats chronically implanted with recording electrodes to investigate the longitudinal impact of a focal cortical lesion on neuronal connectivity between two sensorimotor loop areas (i.e., the primary somatosensory cortex and the premotor cortex) not directly affected by the ischemic lesion in the primary motor cortex. The rationale was to investigate changes undergoing preserved circuitry that can be leveraged to promote functional recovery by means of, among others, neuroprosthetic approaches (Guggenmos et al., 2013; Chiappalone et al., 2022). Specifically, we performed electrophysiological recordings at two post-lesion time points for the assessment of the induced acute and subacute network reorganization (i.e., 7 and 14 days), as well as on animals without induced brain lesion. We extracted the Local Field Potentials (LFPs) and we then employed multiple LFP-based analytical approaches to quantify how sensorimotor network interactions evolved over time during sleep. First, we investigated oscillatory phase locking, as synchrony of neural oscillations across brain regions is thought to facilitate communication between distributed neuronal populations (Palva et al., 2005; Sauseng and Klimesch, 2008; Fell and Axmacher, 2011). Phase synchronization, especially in the low-frequency range, plays a key role during sleep, when neuroplastic processes such as synaptic consolidation and circuit reorganization are most active (Miyamoto et al., 2017; Niethard et al., 2017). In this context, we used the Phase-Locking Value (PLV) to quantify interareal phase synchrony as a proxy for coordinated network activity. Second, we evaluated the organization of brain oscillations across frequency bands, a mechanism thought to support communication across multiple spatiotemporal scales in the brain and quantified by measuring the Phase-Amplitude Coupling (PAC). PAC reflects how the phase of slow oscillations coordinates the amplitude of faster rhythms, allowing for dynamic coordination between local and distributed neural processes.

Our findings reveal a transient increase in low-frequency synchrony and cross-frequency coupling during early post-stroke sleep, followed by partial normalization, reflecting dynamic reorganization of spared cortical circuits during spontaneous recovery.

## 2 Materials and methods

### 2.1 Animals

**Figure 1:**
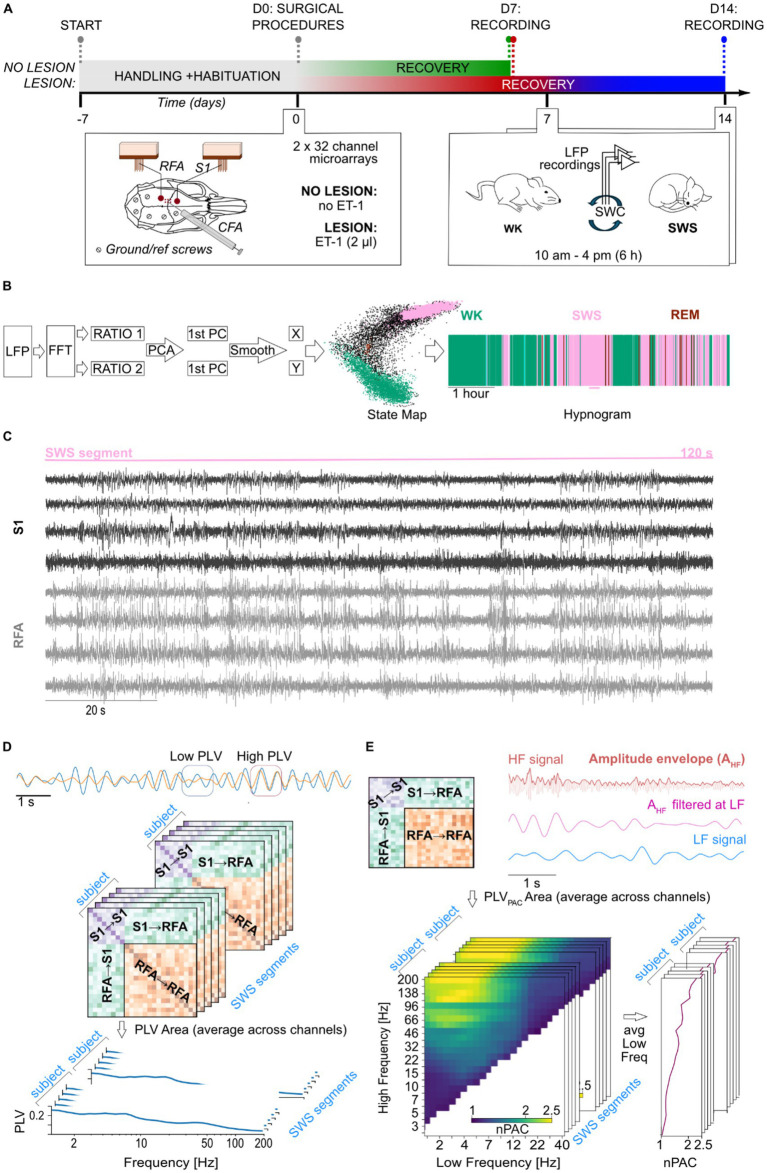
Experimental design and signal processing pipeline. **(A)** Timeline of the experiment that starts with handling of the animals and habituation to the experimental arena, followed by surgical procedures on D0 and recording sessions on D7 for both groups and D14 for lesion animals only. Details of the surgical procedures are shown in the left inset panel, including relative position of implants, microelectrode arrays and ground/reference screws, onto the rat’s skull and the region of the ischemic lesion induced by microinjection of ET-1. On the right inset panel, details of the recording procedure are depicted. **(B)** The steps for the LFP signal processing pipeline for the staging of the SWC are shown on the left, alongside the state map and hypnogram of the same representative animal on the right. **(C)** Representative segment of 2 min of recording of bipolar local field potential (LFP) traces from 8 channels across S1 (black) and RFA (gray) during a segment of slow-wave sleep. **(D)** Schematic representation of phase-locking value (PLV) dynamics. Two example of time series are shown at the top, initially exhibiting low phase synchronization (highlighted by the blue square), followed by an increase in synchronization (highlighted by the orange square). Each matrix, shown in the middle part of the panel, represents phase synchrony between channel pairs during a segment of slow-wave sleep (SWS). Warmer colors indicate higher PLV values, corresponding to stronger phase synchronization. The stacked matrices illustrate multiple SWS segments analyzed for each subject at a given frequency. These were subsequently averaged to obtain mean connectivity spectrum within and between regions of interest: within S1 (S1 → S1), within RFA (RFA → RFA), and between regions (S1 → RFA). **(E)** Phase–amplitude coupling (PAC) analysis. Schematic PAC connectivity matrix showing coupling between channels within S1 (S1 → S1), within RFA (RFA → RFA), and between regions (S1 → RFA and RFA → S1), is shown in top left part of the panel. Top right panel shows representative LFP traces filtered in a high-frequency band (e.g., 40 Hz, magenta) and a low-frequency band (e.g., 2 Hz, blue). The envelope of the high-frequency signal is extracted and filtered at the same low frequency to assess the degree of coupling between the low-frequency phase and high-frequency amplitude. Bottom left represents normalized PAC (nPAC) comodulograms for a selected area (e.g., S1) showing modulation strength across combinations of low (*x*-axis) and high (*y*-axis) frequencies. Yellow regions reflect stronger PAC. The stacked matrices illustrate multiple SWS segments analyzed for each subject. These comodulograms were averaged across the low-frequency axis to derive high-frequency-specific PAC spectrum, as shown in the bottom right panel.

Nine (09) Long Evans male rats (*Rattus norvegicus*), weighting 250–300 g, obtained from Charles River (Milano, Italy), were used in this study. They were housed at the vivarium of IIT’s Animal Facility for the entire duration of the experiment, being kept in a 12-h light–dark cycle (lights on at 7 a.m. and off at 7 p.m.), at an average temperature of 23 °C ± 2 °C, with food and water ad libitum. Animals were equally divided into two groups, controls (NO LESION; *N* = 5) and lesion (LESION; *N* = 4). The sample size was consistent with previous rodent studies combining focal ischemia models and chronic multichannel electrophysiology (Carè et al., 2022). All animals underwent surgical procedure for the implantation of electrodes in two cortical regions of the sensorimotor loop, the Rostral Forebrain Area (RFA) and the primary somatosensory area (S1) (Figure 1A, bottom left inset), as well as 6 h-long electrophysiological recordings (Figure 1A, bottom right inset). Animals in the LESION group were subjected to the induction of an ischemic lesion (see below) in the Caudal Forelimb Area (CFA). All experiments were previously approved by the Italian Ministry of Health (license 513/2022) and were in accordance with international guidelines for the care of animals in research.

### 2.2 Surgical procedure for the implantation of electrodes

To begin the surgical procedure, initial anesthesia was induced with the administration of gaseous isoflurane (5% at 1 lpm) using a dedicated inhalation chamber for the animal. This was followed by the administration of ketamine (intraperitoneal, 80–100 mg/kg; intramuscular, 10–20 mg/kg) and xylazine (intramuscular, 5 mg/kg). After trichotomy of the head, protection of the eyes with ophthalmic ointment, and appropriate aseptic measurements with Betadine and 70% alcohol, animals were positioned in the stereotaxic apparatus. Body temperature was monitored by a rectal probe and controlled by a heat pad using a physiological monitoring system, Physio Suite® (Kent Scientific, Torrington, USA). The sedation status of the animal was continuously monitored by the experimenters, checking for animal twitches through a simple pinch test and, when necessary, maintenance boluses of ketamine (intramuscular, 0.10–0.17 mL) were administered. The skull surface was exposed with a rostro-caudal incision using a scalpel, followed by dissection of the periosteum, and cleaning of the bone with hydrogen peroxide. Furthermore, the cisterna magna was also exposed, and a laminectomy was performed to control brain edema, by allowing excess cerebrospinal fluid drainage. In the sequence, craniectomy was performed to access the primary somatosensory cortex (S1: −1.25 mm AP and +4.25 mm ML, referenced from the Bregma) and the rostral forelimb area (RFA, a pre-motor area: +3.5 mm AP and 2.5 mm ML, referenced from the Bregma) using a drill with a burr bit. Additional six holes were predisposed with a small drill bit into the parietal and intraparietal bones to later insertion or surgical microscrews that served both as reference and grounding, as well as to secure the implant on the animal for the chronic recordings. Dura-mater was carefully removed using a bent hypodermic needle to avoid excessive brain tissue dimpling and electrode bending and damage. Electrodes (32 channel microwire arrays, Microprobes, USA), one for each area, were slowly (0.1 mm/min) inserted into the cortex to a depth of 1.4 mm referenced from the cortical surface. Implantation sites were sealed using low toxicity adhesives for live tissues (Kwik-cast; WPI, Sarasota, USA). The whole assembly, including both electrode arrays, was secured onto the animal’s head with dental auto polymerizing acrylic cement anchored by the bone microscrews. Finally, the skin opening was sutured, the animals were cleaned, received antibiotics and anti-inflammatory drugs, and remained under observation in a heated cage until they were fully awake and moving. All animals were left to rest for 7 days before beginning experiments. During this period, animals were monitored daily for signals of discomfort or insufficient healing and were treated with analgesics, antibiotics, and anti-inflammatory drugs on the second and third days.

### 2.3 Induction of the ischemic stroke model

Animals in the LESION group were submitted to the ischemic stroke model by microinjection of Endothelin-1 (ET-1), a potent vasoconstrictor (Frost et al., 2006). During the surgical procedure, before the insertion of electrode arrays, six small holes were drilled in the skull centered around the CFA, equivalent to human primary motor cortex (combining coordinates at AP: +1.5 mm, +0.5 mm, and −0.5 mm; ML: +3.5 mm and +2.5 mm, referenced from Bregma). ET-1 was then loaded into a Hamilton syringe which was lowered 1.2 mm into brain tissue. A volume of 0.33 mL per hole (thus a total of 2 mL) was injected, divided into three steps of 0.11 mL gradually administered at a 3 nL/s rate; sessions being interspaced by 1 min. This guaranteed a seamless absorption of the vasoconstrictor agent volume without damage by tissue displacement (Carè et al., 2022).

### 2.4 Electrophysiological recordings and signal pre-processing

In order to lower stress levels, avoid excessive exploratory behavior, and maximize natural expression of sleep–wake cycle, all animals were handled by the same experimenter for 15 min and left to explore the controlled environment (i.e., a squared arena with a base of 60 × 60 cm, and 60 cm height) for another 15 min, for 5 days, twice a day, before surgery (Figure 1A, experimental timeline). In the day of the recording session, animals were lightly anesthetized with gaseous isoflurane (5% at 1 lpm) for cleaning of electrode array contacts and for the placement of recording headstages and cables. Animals were then placed in the arena and cables connected to the electrophysiological setup. The system was composed of headstages (equipped with RHS 2000 series chips; Intan Technologies LCC, Los Angeles, USA) for amplification (192 V/V gain), analog bandpass filtering (1 Hz–7.5 kHz), and digitalization (16 bits resolution at 25 kHz), connected by an SPI cable to the controller for PC communication via USB. Wideband recording signals were then low-pass filtered at 300 Hz and downsampled to 1 kHz using the NigeLab pipeline for MATLAB developed at our lab,^1^ already adopted in previous studies (Carè et al., 2022). Furthermore, a bipolar referencing method was chosen, and 50 Hz line noise and its harmonics were excluded with a band-stop FIR filter achieving 53 dB suppression with 1 Hz band-stop widths using custom-made Python functions specifically developed for this study. Recordings were carried out at least 1 h after termination of gaseous anesthesia for the proper metabolization of the drug, from 10 a.m. to 4 p.m. (±30 min). The NO LESION animals were subjected to a single recording session, while LESION animals underwent two recording sessions at 7 and 14 days post lesion. Specifically, recordings were scheduled to allow full recovery from surgery and the acute ischemic phase (day 7) as well as to capture the early subacute stage of stroke recovery (day 14), when reorganization processes begin (Boboc et al., 2023).

### 2.5 SWC scoring

Sleep stages were automatically detected by clustering points in a state map derived from spectral content ratios (Gervasoni et al., 2004) (Figure 1B). Briefly, after removing channels plagued by movement (characterized by constant drastic increases of amplitude with signal clipping), the power spectrum of 2-s windows from LFPs recordings was computed separately for each channel at 1-s intervals (with 50% overlap). Two spectral power ratios were then calculated: ratio 1 [(0.5–20 Hz)/(0.5–55 Hz)] and ratio 2 [(0.5–4.5 Hz)/(0.5–9 Hz)]. Principal Component Analysis (PCA) was applied to these ratios, and the resulting time series of the two first principal components (1st PC of ratio 1 and 1st PC of ratio 2) were smoothed using a 20-s Hanning window. The smoothed PC values were plotted along the *X* (ratio 2) and *Y* (ratio 1) axes of the state map, respectively. Clusters in this map were then identified and assigned to one of the three major sleep–wake cycle (SWC) states, wakefulness (WK), slow-wave sleep (SWS), or rapid eye movement (REM) sleep, based on their position, following the method described in the literature (Gervasoni et al., 2004). The window size of 2 s was selected to enable the calculation of the lower limit of band ratios, while the band ranges were empirically determined and repeatedly shown in the literature to effectively separate sleep states in the state map (Gervasoni et al., 2004; Dzirasa et al., 2006; Blanco et al., 2015; Réboli et al., 2022). For the analyses of power, phase synchronization, and phase–amplitude coupling (described below), we empirically selected SWS segments of 120 consecutive seconds that were artifact-free (e.g., movement, chewing, or environmental artifacts) and contained no bad channels, defined as those with baseline drift or poor signal-to-noise ratio, after visual inspection. A total of 48 segments were included for the NO LESION group (mean ± SD = 10 ± 3 segments per animal), 91 segments for the 7-day post-LESION group (23 ± 2 segments per animal), and 94 segments for the 14-day post-LESION group (23 ± 5 segments per animal). All metrics considered for this work, and described below, were computed for each segment and later averaged over them. Figure 1C shows an example of raw LFP traces with bipolar referencing from a 120-s segment of SWS, including four channels each from the S1 and RFA areas.

### 2.6 Power spectral density

To estimate spectral power during slow-wave sleep in animals, we computed the Power Spectral Density (PSD) by employing Welch’s method with a sampling rate of 1,000 Hz and a Hanning window length of 4,096 samples, corresponding to a frequency resolution of approximately 0.24 Hz. A 50% overlap was applied between the windows. PSD estimates were then averaged across channels within each region of interest, i.e., S1 and RFA.

### 2.7 Phase locking value

To investigate both local and inter-areal phase synchronization, we used the Phase-Locking Value (PLV). PLV is derived as the absolute value of the complex PLV (cPLV) (Palva et al., 2018; Arnulfo et al., 2020). We band-pass filtered the LFP data using 43 Morlet wavelets (width *m* = 7.5) with center frequencies ranging from 1.2 to 200 Hz and we then computed the cPLV between two signals x and y, defined as:


$${\text{CPLV}}_{x,y}=\frac{1}{K}\sum_{k=1}^{K}\frac{x\prime (k){y\prime }^{*}(k)}{|x\prime (k)\|y\prime (k)|}$$

Where x’(t) and y’ represent the complex wavelet coefficients of the signals at a given frequency, K represents the total number of samples of the entire signal, and * denotes the complex conjugate. The PLV provides a scalar measure between 0 and 1, with 1 indicating complete phase synchronization and 0 indicating complete desynchronization. An example of how PLV is computed is shown in Figure 1D.

Synchronization matrices were further analyzed using graph-theoretical metrics. Specifically, eigenvector centrality (EVC) was computed to estimate each channel’s centrality within the functional connectivity network during slow-wave sleep. These metrics were aggregated at the subject level and subsequently correlated with total sleep duration to investigate potential associations between network organization and sleep stability (*cf.* Statistical analysis section).

### 2.8 Phase amplitude coupling

Phase-amplitude coupling (PAC) provides information about the correlation between the phase of the slower oscillation and the amplitude of the faster one (Vanhatalo et al., 2004; Siebenhühner et al., 2020). PAC was computed between selected low-frequency (LF) and high-frequency (HF) pairs as follows:


$$\mathrm{PA}C_{x,y}=\frac{1}{K}|\sum_{k=1}^{K}e^{i(\theta_{x,\mathrm{LF}}-\theta_{y,\mathrm{HF},\mathrm{LF}}^{\mathrm{env}})}|$$

Where $\theta_{x,\mathrm{LF}}$ represents the phase of a given low frequency signal and $\theta_{y,\mathrm{HF},\mathrm{LF}}^{\mathrm{env}}$ denotes the amplitude envelope of high frequency signal filtered at the same frequency of the LF. Like the PLV, the PAC provides a measure ranging from 0 to 1, with 1 denoting full phase amplitude interaction and 0 indicating no interaction. For our analysis we considered the normalized PAC (nPAC = PAC_PLV,obs_/PAC_PLV,sur_), representing PAC above the null hypothesis level. The following low-frequency (LF) bands were used for the computation of phase–amplitude coupling (PAC): [1.2, 2.4, 3.7, 5.9, 8.6, 13.2, 19.5, 29.5, 47.3, 68.1] Hz. For each LF component, corresponding high frequency (HF) bands were generated by multiplying the LF value by a set of predefined ratios (HF = LF*ratio). The ratio considered here are: [2, 3, 4, 5, 6, 7, 8, 9, 10, 11, 14.06, 17.96, 22.96, 29.34, 37.49, 47.91, 61.23, 78.25, 100, 110, 149.11, 202.13]. The nPAC were discarded if the resulting HF component exceeded 200 Hz. To facilitate readability and matrix visualization, nPAC results were projected from the low-frequency-to-ratio format into a low-frequency-to-high-frequency space. Subsequently, nPAC values were averaged across all low-frequency bands for each high-frequency band, yielding a mean nPAC value for each high-frequency. We also differentiated between local and inter-areal nPAC. Intra-area nPAC measures phase-amplitude interactions within electrodes in the same brain region, while inter-areal nPAC evaluates interactions between the phase of low frequencies in one electrode and the amplitude of high frequencies in another electrode located in a different region. An example of how nPAC is computed is shown in Figure 1E.

### 2.9 Statistical analysis

To assess the validity of our PLV and PAC computation, we compared them to equivalent analyses performed on surrogate data. We designed surrogate data to disrupt inter-channel correlations while preserving the temporal autocorrelation structure of the original signals. Specifically, for each pair of narrow-band time series, we selected a random time point k uniformly along the signal and split each signal into two segments. A surrogate signal was then constructed by recombining mismatched segments from the two channels:


$$x_{\text{surr}}=[x_{1}(k,\ldots ,t),x_{1}(1,\ldots ,k)]$$

This procedure maintains the intrinsic temporal structure of each signal, while interactions between the signals are disrupted. Surrogate analysis was applied with two distinct purposes depending on the metric. For PLV, surrogate levels were included in the main graphs of the phase synchronization spectra to provide a visual reference for the noise level in the data, whereas for PAC, surrogate analysis was used to normalize the PAC values, as described in detail in the corresponding analysis section (cf. Phase amplitude coupling section).

Due to substantial data heterogeneity, we employed multiple statistical approaches to test for differences in PLV and PAC across frequency bands and regions of interest between experimental conditions (i.e., NO LESION, 7 days post-lesion, and 14 days post-lesion). Before performing all comparisons, we first assessed the normality of the data using the Shapiro–Wilk test, which confirmed that the data were normally distributed. To compare the no lesion group with animals at 7 days post-lesion, we used the Mann–Whitney *U* test to assess differences in central tendency, and the Fligner–Killeen test with the Coefficient of Variation (CV) to assess differences in variability. For the within-subject comparison between 7 and 14 days post-lesion in the same animals, we employed a paired *t*-test for paired differences in central tendency, and a permutation test on the logarithm of the variance, alongside the CV, to evaluate changes in variability. These tests were chosen considering the unpaired and paired conditions of the former and latter comparisons, respectively. In addition, effect sizes were computed to provide a measure of the magnitude of observed differences independently of sample size, with Cohen’s d being used for pairwise comparisons. Moreover, linear mixed-effects models (*lme4* package in R (Bates et al., 2015) were employed to test for correlations between sleep duration and PLV, as well as for changes in these correlations across experimental time points (*emmtrends* function, *emmeans* library in R (Lenth and Piaskowski, 2025). We chose these two metrics as evident tokens of behavior and brain activity, given the well-established notion that the architecture of SWC (including its duration)—and the neuroplastic phenomena supported by it—are largely dependent on the proper coordination and synchronization of neural oscillations (PLV eigenvalue centrality). For all statistical tests, *p*-values were corrected for multiple comparisons across frequencies using the Benjamini–Hochberg procedure (*α* = 0.05). Frequency bands are referred to here by their canonical names: delta (0.5–4 Hz), theta (4–8 Hz), alpha (8–12 Hz), beta (12–30 Hz), and gamma (30–100 Hz).

## 3 Results

### 3.1 Spectral power dynamics in S1 and RFA during SWS following cortical lesion

**Figure 2:**
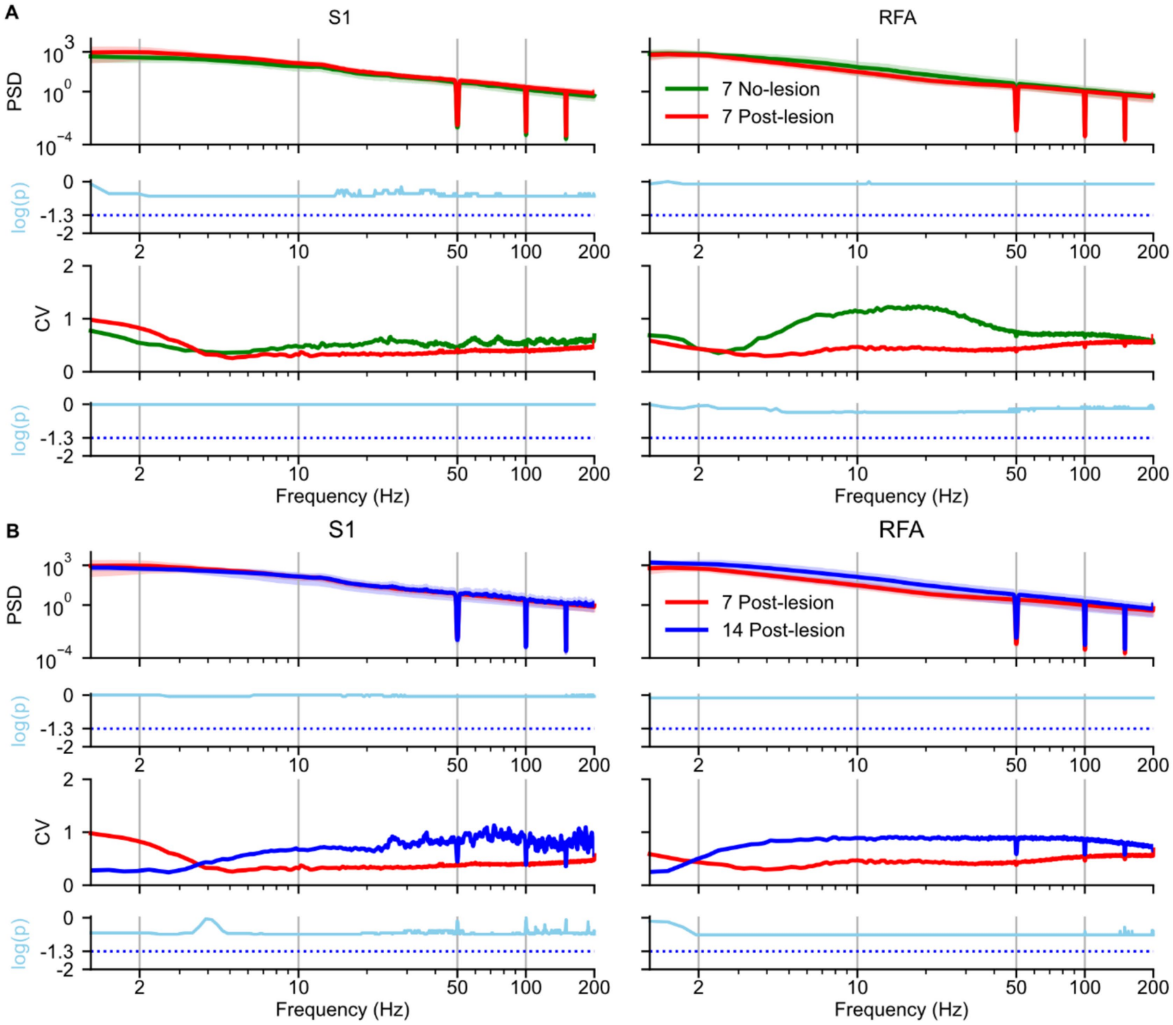
Effects of ischemic lesion on spectral power during slow-wave sleep. Spectral analyses and statistical comparisons of power spectral density (PSD) during slow-wave sleep are shown for: **(A)** the no-lesion group vs. lesion group recorded 7 days post-lesion, and **(B)** the lesion group at 7 vs. 14 days post-lesion. Each panel is arranged in a 4 × 2 grid. Rows represent: (1st row) group-averaged PSD; (2nd row) log-transformed *p*-values from statistical tests on PSD distributions; (3rd row) coefficient of variation (CV), and (4th row) log-transformed p-values from tests comparing variance. Columns represent brain regions S1 and RFA. In panel A, green lines represent the no-lesion group (*n* = 5), and blue lines represent the lesion group at 7 days (*n* = 4). In panel B, blue and red lines represent lesion recordings at 7 and 14 days, respectively (*n* = 4). In both panels A and B in the first row, spectra are shown as solid lines (group means) with shaded areas representing 97% confidence intervals estimated via bootstrap resampling (*n* = 1,000). The second row shows log-transformed *p*-values (cyan); statistical comparisons used Mann–Whitney *U* tests in panel A and paired *t*-test in panel B. All *p*-values were corrected for multiple comparisons across frequencies using the Benjamini–Hochberg procedure (*α* = 0.05), the cyan dotted line represents the *p*-value = 0.05. The third row (panels A and B) shows the coefficient of variation (CV) across animals, reflecting inter-subject variability. The fourth row displays results from Fligner’s test (panel A) and a permutation-based test for variance differences (panel B), also corrected for multiple comparisons, the cyan dotted line represents the *p*-value = 0.05.

To assess the impact of ischemic lesions on neural oscillatory activity during SWS, we analyzed the PSD of LFPs recorded from electrodes implanted in S1 and RFA (see Methods), Figure 2. Comparisons were made between the NO LESION group and the LESION group at 7 days post-lesion (Figure 2A), as well as between recordings at 7 and 14 days post-lesion (Figure 2B). In S1 (Figure 2A, left) the PSD at 7 days post-lesion did not differ significantly from the NO LESION group (*p* = 0.29), although a medium effect size was observed (Supplementary Figure 1A, left). Intersubject variability appeared elevated in the delta band [1–4 Hz], as reflected by a higher coefficient of variation (CV) in the 7-day post-lesion group compared to controls (i.e., NO LESION). However, this difference was not statistically significant (*p* = 0.98), and the corresponding log-ratio of variances (i.e., lnVR = 1.98; Supplementary Figure 1A, left). In contrast, RFA (Figure 2A, right) showed a reduction in PSD between 4–50 Hz frequency range at 7 days post-lesion. Although this change was not statistically significant (*p* = 0.80), the effect size was medium (Supplementary Figure 1A, right), This reduction was also reflected in the CV, which showed lower values in the same frequency range after lesion, even if without statistical significance (*p* = 0.5). The log-ratio of variances was −1.2 (Supplementary Figure 1A, right).

Between 7 and 14 days post-lesion, PSD in S1 (Figure 2B, left) remained stable (*p* = 0.90), with a negligible effect size (Supplementary Figure 1B, left). The CV was lower at 14-day than at 7-day in the delta band [1–4 Hz], although this difference was not statistically significant (*p* = 0.36) with lnVR of −2.53 (Supplementary Figure 1B, left). In RFA (Figure 2B, right), PSD across the full spectrum showed an increase at 14 days post-lesion (*p* = 0.77) with medium effect size (Supplementary Figure 1B, right). This increase was accompanied by a rise in CV, although no statistically significant differences were found in variability (*p* = 0.26) and with lnVR of 2.42 (Supplementary Figure 1B, right).

### 3.2 Functional connectivity between S1 and RFA during SWS after ischemic lesion

**Figure 3:**
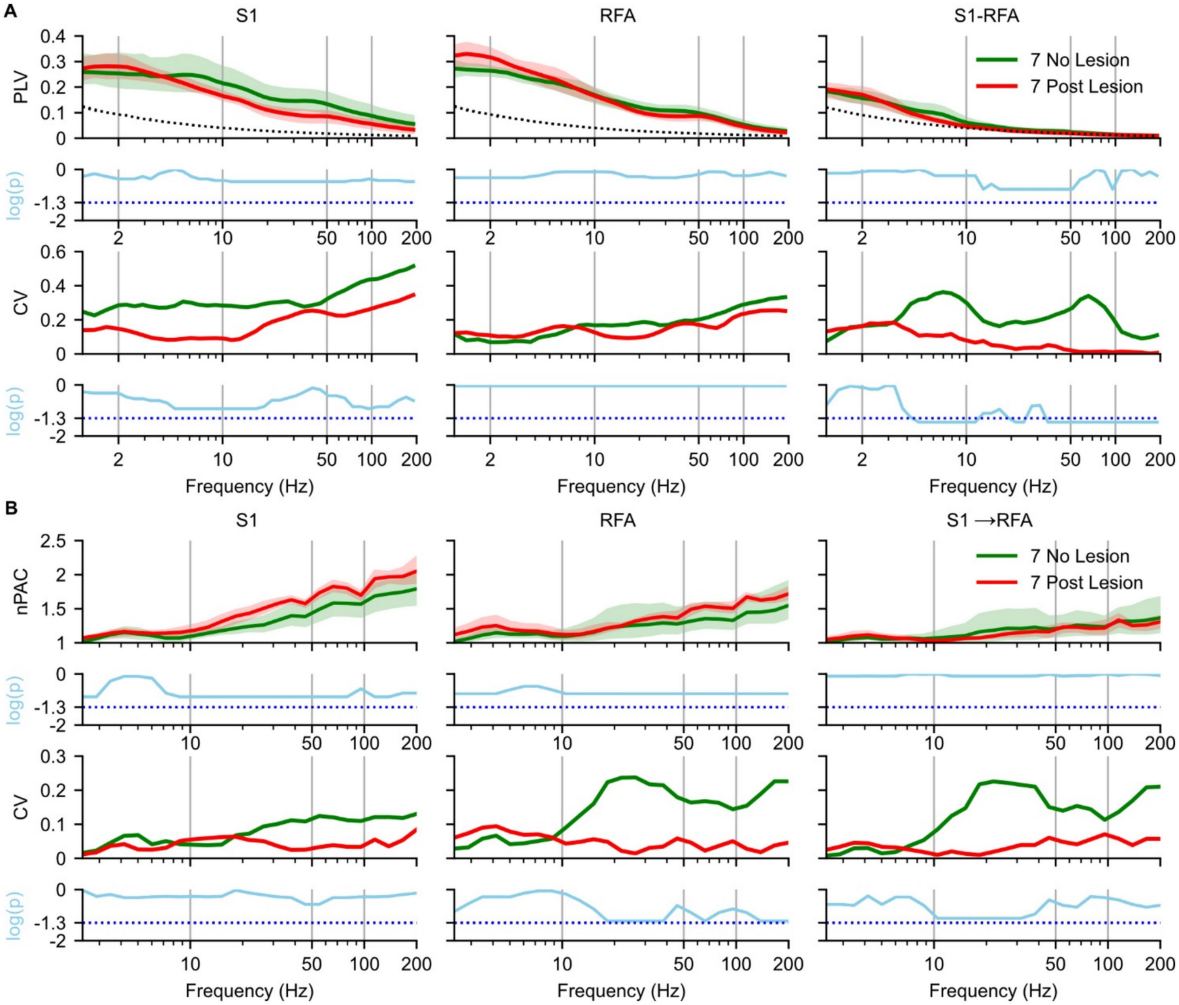
Effects of ischemic lesion on brain connectivity during slow-wave sleep. Spectral analyses and statistical comparisons of phase-locking value (PLV; panel A) and phase–amplitude coupling (PAC; panel B). Each panel is organized in a 4 × 3 grid, with rows representing analysis type: (1st row) spectral profile, (2nd row) p-values from pre- vs. post-lesion Mann–Whitney *U* test, (3rd row) coefficient of variation (CV), and (4th row) *p*-values from pre- vs. post-lesion Fligner’s test, and columns corresponding to brain regions and inter-areal connections. For PLV **(A)**, columns represent S1 (left), RFA (middle), and connectivity between the two regions (right). For PAC **(B)**, columns represent S1 (left), RFA (middle), directional coupling from S1 to RFA (right). Green indicates no lesion group recorded at day 7 post implant (*n* = 5 animals), and red indicates recordings at 7 days post-lesion (*n* = 4 animals) and the dotted black line represents the mean of surrogate data. In the first row, spectra are shown as solid lines (group means) with shaded areas representing 97% confidence intervals estimated via bootstrap resampling (*n* = 1,000). The second row shows log-transformed *p*-values (cyan) from Mann–Whitney *U* tests comparing no- and post-lesion conditions values across frequencies, corrected for multiple comparisons using the Benjamini–Hochberg correction (*α* = 0.05), the cyan dotted line represents the *p*-value = 0.05. The third row shows the coefficient of variation (CV) across animals, reflecting inter-subject variability. The fourth row presents log-transformed *p*-values from Fligner’s test assessing group differences in variance, also corrected using the Benjamini–Hochberg correction (*α* = 0.05), the cyan dotted line represents the *p*-value = 0.05.

Next, we assessed whether ischemic lesions affected phase synchronization during SWS (Figure 3), by comparing the 7 day NO LESION and the 7 day LESION groups. In S1 (Figure 3A, left), within the delta band [1–4 Hz], PLV was higher post-lesion than in the NO LESION condition (*p* = 0.52) with a small effect size (Supplementary Figure 2A, left). In contrast, at frequencies above the delta band, PLV of LESION group began to decrease (*p* = 0.34), showing a large effect size (Supplementary Figure 2A, left). This reduction was accompanied by a decrease in the CV compared to the NO LESION group (*p* = 0.25) with a mean lnVR of −1.94 (Supplementary Figure 2A, left). At 7 days post lesion, in RFA (Figure 3A, middle), an increase in the delta band was observed (*p* = 0.53), with a large effect size (Supplementary Figure 2A, middle). CV values increased, though not significantly (*p* = 0.92), with a mean lnVR of 0.8 (Supplementary Figure 2A, middle). Inter-areal PLV between S1 and RFA (Figure 3A, right) showed increased delta synchronization post-lesion, even if not statistical (*p* = 0.82) with small effect size (Supplementary Figure 2A, right), and decreased theta [4–8 Hz] -band PLV (*p* = 0.56) with large effect size (Supplementary Figure 2A, right). This was accompanied by a significant reduction in variability, as indicated by decreased CV and a significant Fligner’s test result (*p* = 0.035), with a mean lnVR of −2.1 (Supplementary Figure 2A, right). PLV values above the theta band dropped to near-surrogate levels in both LESION and LESION conditions (Figure 3A, right).

For PAC, the spectrum in S1 (Figure 3B, left), RFA (Figure 3B, middle), and directional coupling from S1 → RFA (Figure 3B, right) showed similar patterns in NO LESION and 7 days post-lesion up to 20 Hz (*p* = 0.27, 0.20, and 0.81, respectively), with strong effect sizes in S1 and RFA (Supplementary Figure 2B, left and middle). In both S1 (Figure 3B, left) and RFA (Figure 3B, middle), PAC above 20 Hz was higher post-lesion (*p* = 0.15 and 0.17, respectively) with large effect (Supplementary Figure 2B, left and middle). The CV in the LESION group decreased in RFA and in S1 → RFA (Figure 3B, middle and right). However, no frequency bands showed statistically significant differences in variance after correction for multiple comparisons, despite trends approaching significance (*p* = 0.10) with a lnVR of 3.36 and lnVR of 3, respectively, (Supplementary Figure 2B, middle and right). We also evaluated the modulation from RFA to S1 (see Supplementary Figure 4A); however, no notable differences were observed between the two groups.

### 3.3 Longitudinal effects of post-lesion recovery on functional connectivity

**Figure 4:**
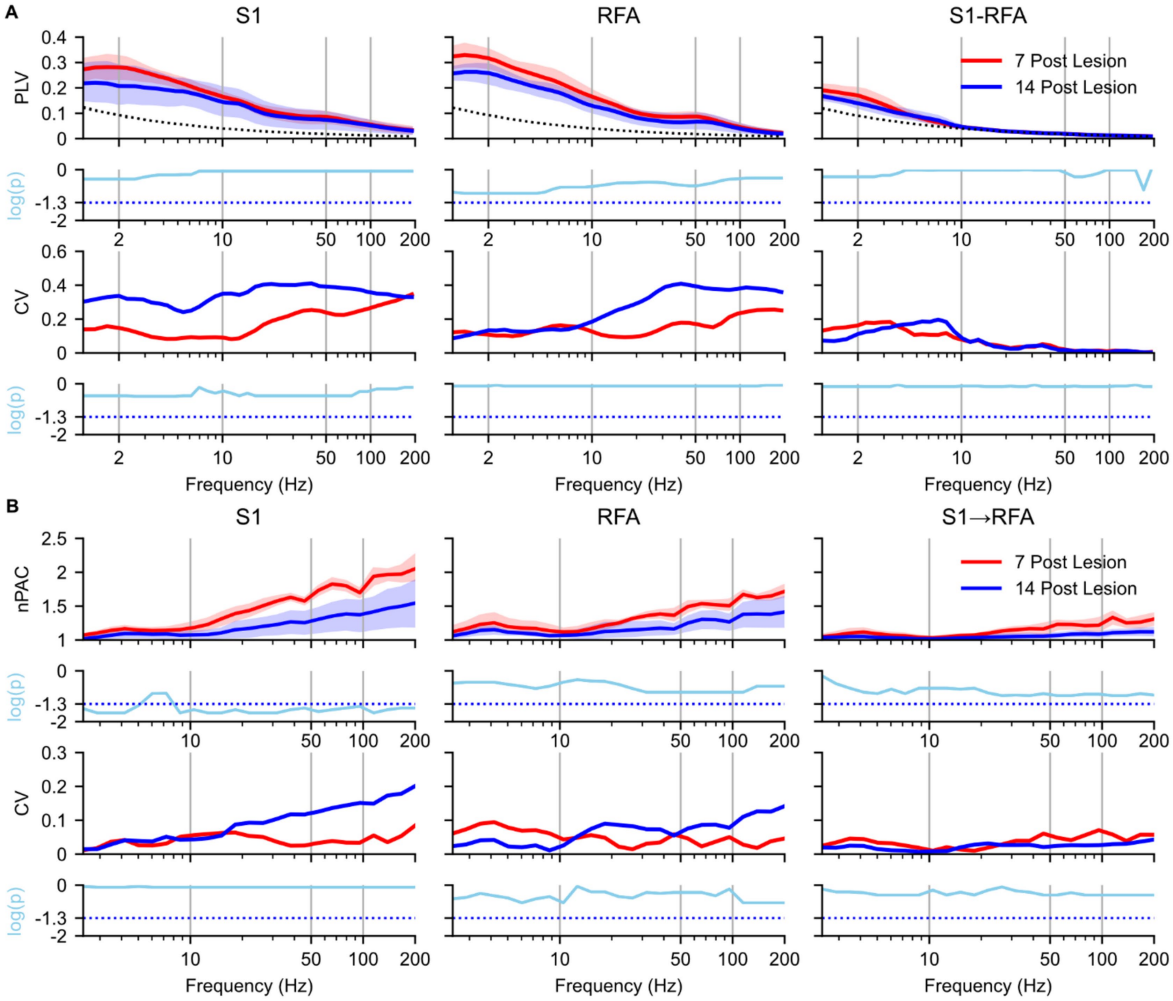
Effects of natural recovery after ischemic lesion on brain connectivity during slow-wave sleep. Spectral analyses and statistical comparisons of phase-locking value (PLV; panel A) and phase–amplitude coupling (PAC; panel B). Each panel is organized in a 4 × 3 grid, with rows representing analysis type: (1st row) spectral profile, (2nd row) *p*-values from 7- vs. 14 days post-lesion a paired *t*-test, (3rd row) coefficient of variation (CV), and (4th row) *p*-values from 7- vs. 14 days permutation test on the logarithmic differences in variance, and columns corresponding to brain regions and inter-areal connections. For PLV **(A)**, columns represent S1 (left), RFA (middle), and connectivity between the two regions (right). For PAC **(B)**, columns represent S1 (left), RFA (middle), directional coupling from S1 to RFA (right). Red indicates 7 days post-lesion group (*n* = 4 animals) while blue represents 14 days post-lesion group (*n* = 4 animals) and the dotted black line represents the mean of surrogate data. In the first row, spectra are shown as solid lines (group means) with shaded areas representing 97% confidence intervals estimated via bootstrap resampling (*n* = 1,000). The second row shows log-transformed *p*-values (cyan) from paired *t*-test comparing 7- vs. 14 days post-lesion conditions values across frequencies, corrected for multiple comparisons using the Benjamini–Hochberg correction (*α* = 0.05), the cyan dotted line represents the *p*-value = 0.05. The third row shows the coefficient of variation (CV) across animals, reflecting inter-subject variability. The fourth row presents log-transformed *p*-values from permutation test assessing group logarithmic differences between variance, also corrected using the Benjamini–Hochberg correction (*α* = 0.05), the cyan dotted line represents the *p*-value = 0.05.

To assess how phase synchronization evolved over time, we compared PLV and PAC between 7 and 14 days post-lesion during SWS within the LESION group (Figure 4). In S1 (Figure 4A, left), PLV in both the delta and theta bands resulted reduced at 14 days compared to the 7 days post-lesion (*p* = 0.77), with a large effect size (Supplementary Figure 3A, left). At 14 days, the CV in S1 increased (*p* = 0.4), with a mean lnVR of 1.2 (Supplementary Figure 3A, left). A similar pattern was observed in in RFA (Figure 4A, middle), with reductions of the PLV in the delta and theta-band at 14 days (*p* = 0.33), and a large effect size (Supplementary Figure 3A, middle). The CV above 10 Hz also increased at 14 days (*p* = 0.82), with a mean lnVR of 1.1 (Supplementary Figure 3A, middle). Inter-areal PLV between S1 and RFA (Figure 4A, right) showed a decrease in delta-band synchronization (*p* = 0.73) with a large effect size (Supplementary Figure 3A, right), and an increase in theta-band PLV at 14 days post-lesion (*p* = 0.9), with a medium effect size (Supplementary Figure 3A, right). In line with these spectral changes, the CV in inter-areal synchronization decreased in the delta band (*p* = 0.77) with a mean lnVR of −1.2 (Supplementary Figure 3A) and increased in the theta band (*p* = 0.8) with a mean lnVR of 0.86 (Supplementary Figure 3A). PLV values at frequencies above the theta band dropped to near-surrogate levels at both experimental groups.

As regards the PAC analysis, in S1 (Figure 4B, left), within the delta band and for frequencies above 10 Hz, PAC values were significantly higher at 7 days post-lesion compared to 14 days post-lesion (*p* = 0.04 and *p* = 0.018, respectively), with a large effect size (Supplementary Figure 3B). The CV in S1 above 10 Hz increased at 14 days, although the changes did not reach statistical significance in any frequency band (*p* = 0.08) and a mean lnVR of −1.58 (Supplementary Figure 3B, left). PAC spectral profiles in RFA(Figure 4B, middle) and in the S1 → RFA direction (Figure 4B, right) remained unchanged between 7 and 14 days post-lesion for frequencies up to 20 Hz (*p* = 0.23 and *p* = 0.75, respectively), despite large effect sizes (Supplementary Figure 3B, middle and right). Above 20 Hz, however, a reduction in PAC was observed at 14 days in both regions. PAC showed *p*-values approaching the significance threshold (*p* = 0.075), along with large effect sizes (Supplementary Figure 3B, middle and right).). CV in the RFA and S1 → RFA direction remained almost stable over time for both groups (*p* = 0.08) and a lnVR of −1.5 (Supplementary Figure 3B, middle and right). Finally, modulation from RFA to S1 was also evaluated, but no notable differences were observed between the groups (see Supplementary Figure 4B).

### 3.4 Sleep duration dynamically modulates phase connectivity in both areas after lesion

**Figure 5:**
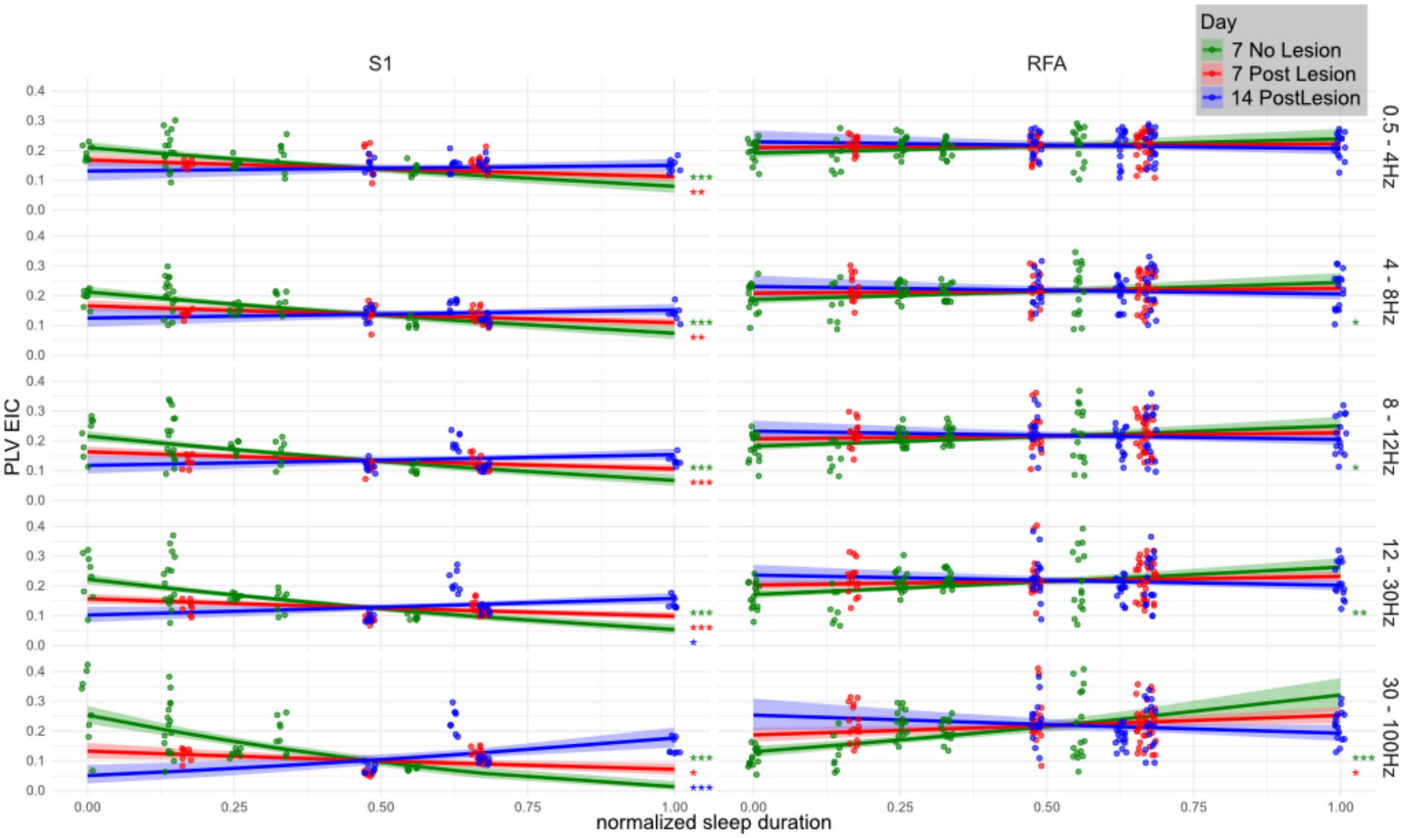
Relationship between eigenvector centrality of PLV networks and SWS duration across frequency bands, brain regions, and lesion stages. Linear mixed effects model showing the correlation between total (normalized) SWS duration (*x*-axis) and the eigenvector centrality of the phase locking value (*y*-axis) across animals (*n* = 4 per group) in three conditions: healthy (Day 7 No Lesion; green), 7 days post-lesion (red), and 14 days post-lesion (blue). Panels are organized by brain region (S1 and RFA) and frequency band (in Hz, labeled on the right). Solid lines indicate model estimated trends, and shaded areas represent 95% confidence intervals. Overlaid scatterplots show individual values for eigenvector centrality of PLV (i.e., computed over channel pairs). Eigenvector centrality was computed per region and frequency band then square-root transformed before model fitting, revealing a mildly quadratic relationship. Trend significance was tested against the null hypothesis of zero slope using the emtrends() function from the emmeans R package applied to linear mixed-effects models. Asterisks denote significance levels (*p < 0.05; **p < 0.005; ***p < 0.0005), with *p*-values corrected for multiple comparisons using the Benjamini–Hochberg method.

To determine how SWS duration shapes functional network organization after a focal CFA lesion, we fitted linear mixed-effects models testing the slope of normalized SWS duration against square-root–transformed eigenvector centrality of PLV networks, Figure 5. Trends were estimated with *emtrends* and significance was corrected for multiple comparisons by the Benjamini–Hochberg method. Full statistics are provided in Supplementary Table 1.

The association between sleep duration and network centrality displayed robust frequency- and region-specific changes following the lesion. Across both regions, centrality estimates in the gamma band during SWS were positively associated with sleep duration in healthy animals (NO LESION). However, in lower frequency bands, this relationship was markedly negative in S1 and close to neutral in RFA, indicating a functional divergence between the two areas even in absence of the injury.

Focusing first on S1, where effects were strongest and most consistent, a pronounced inverse relationship between sleep duration and centrality was observed at baseline across delta, theta, alpha, and beta bands (e.g., delta: *t* = −6.06, *p* < 0.001; gamma: *t* = −8.81, *p* < 1e−15). After the lesion, this association weakened significantly, as shown by positive and statistically significant day-to-day contrasts. For instance, from the NO LESION to the LESION group at day 7, the delta band slope changed by +0.10 (*t* = −4.34, *p* = 0.00012), and the gamma band by +0.30 (*t* = −8.52, *p* = 9.1e−15), clearly indicating a reduction in the negative association.

In RFA, the lesion also disrupted sleep–PLV associations, though effects were more subtle and frequency-dependent. In NO LESION condition, PLV centrality in RFA exhibited a mild positive association with sleep duration, increasing with frequency (e.g., beta: *t* = 4.70, *p* < 0.001; gamma: *t* = 5.95, *p* = 7.5e−8). In presence of the lesion, however, these associations largely vanished, especially at lower frequencies, and only partially recovered by Day 14. The day-to-day contrasts show this flattening clearly. From the NO LESION to the LESION at Day 7 condition, the slope in the alpha band decreased significantly (*t* = 2.80, *p* = 0.015), as did theta (*t* = 2.34, *p* = 0.034) and beta (*t* = 3.88, *p* = 0.002). Gamma again showed the strongest disruption (*t* = 5.02, *p* = 4.1e−6).

In S1, the association between normalized sleep duration and PLV eigenvector centrality remained altered between Day 7 and Day 14 post-lesion, showing no signs of recovery across any frequency band. At lower frequencies, including delta, theta, and alpha, the relationship remained negative, with significant slope differences between the two time points (delta: *t* = −4.34, *p* = 1.2 × 10^−4^; theta: *t* = −5.10, *p* = 2.1 × 10^−5^; alpha: *t* = −5.99, *p* = 4.0 × 10^−6^). Similarly, in the beta band the lesion-induced disruption persisted over time (*t* = −7.98, *p* = 1.7 × 10^−7^), while in the gamma range the centrality remaining negatively associated with sleep duration even 14 days post lesion (*t* = −8.52, *p* = 9.1 × 10^−15^).

In contrast, RFA exhibited frequency-dependent effects. At lower frequencies, in the delta band, the slope differences between Day 7 and Day 14 did not reach statistical significance. However, at higher frequencies the lesion effects remained evident: in the alpha band, the association between sleep duration and centrality was significant (*t* = 2.34, *p* = 0.034), while in the beta band the positive baseline relationship was reduced (*t* = 3.88, *p* = 0.002). The gamma band showed a significant disruption persisting up to Day 14 (*t* = 5.02, *p* = 4.1 × 10^−6^).

## 4 Discussion

In this study, we investigated the impact of a focal ischemic lesion to CFA on neural connectivity between S1 and RFA in rats. LFPs acquired during SWS revealed that spectral power in S1 remained largely stable, whereas RFA displayed a moderate, though non-significant, reduction across 4–50 Hz at 7 days post-lesion, followed by partial recovery at 14 days. Moreover, PLVs in the delta band (~1 to 4 Hz) increased in S1, RFA, and the S1 → RFA connection at 7 days and returned toward baseline levels by day 14. Similarly, PAC below 20 Hz was transiently enhanced at 7 days post lesion and declined thereafter, with this increase being directionally selective and stronger from S1 → RFA than from RFA → S1. Together, these findings indicate a brief sub-acute window of low-frequency hyper synchronization and enhanced cross-frequency interactions in spared cortices, followed by a trend toward physiological re-normalization.

This pattern of complex modifications, which are polyphasic across time and heterogenous between regions, corroborates the notion of cortical reorganization following injury. This is because the investigated areas, RFA and S1, are not the direct targets of the ischemic lesion performed here, even though they integrate the sensorimotor loop, in which CFA (lesioned area) plays a major role. Thus, instead of a straightforward display of impaired activity and circuit disconnection, it is highly plausible that the electrophysiological biomarkers assessed would reflect spontaneous network reorganization supported by acute (7 days Post Lesion) and sub-chronic (14 days Post Lesion) homeostasis mechanisms. Additionally, individual characteristics of each area may explain the results. RFA is known for its high degree of plasticity in both healthy and dysfunctional conditions, a key feature for allowing a versatility of motor strategies during learning or recovery (Nishibe et al., 2010; Okabe et al., 2016). Moreover, RFA and CFA have strong interconnections for the support of the motor function (Kreis et al., 2022). On the other hand, S1, while also plastic, may follow a different reorganization trajectory as it mainly drives sensorial inputs to motor areas, receiving only considerably weaker feedback for fine tuning of sensory function (Guzzetta et al., 2007), which could explain its more stable power profile yet marked connectivity alterations.

In this vein, spectral content results (i.e., trend of decreased spectral power in RFA 7 days post lesion and analogously a recovery after 14 days) may represent an initial and faint reflection of this reorganization, expressing the acute loss of two-way coordination with the impaired CFA followed by recovery of activity levels over a longer time frame. Similar reductions in neuronal firing and glucose uptake have been reported in spared motor areas early after stroke, with recovery paralleling behavioral improvement (Cramer, 2004). The partial rebound we observed at 14 days might therefore signal homeostatic upregulation or recruitment of alternative excitatory pathways. Yet, these results were not statistically significant and, while this may be due to basic homeostasis mechanisms coming into action, further testing of this hypothesis is certainly required.

On the other hand, the connectivity assessment of our study provided a clearer picture of the changes induced by the lesion. PLV analysis revealed a transient increase in delta-band synchronization between S1 and RFA, with values at 14 days post-lesion trending back toward those observed in non-lesioned animals. Slow oscillations, especially during SWS, are a hallmark of network and cellular processes that culminate in calcium signaling leading to the expression of immediate early genes and consequent synaptic remodeling (Blanco et al., 2015; Timofeev and Chauvette, 2017), supporting network rewiring. Thus, the initial increase we found on day 7 and, later, the normalization by day 14 after the lesion likely represent a shift from an initial permissive plastic state to a more refined circuit configuration afterwards. Another key finding supporting this rationale was the alteration of PAC, which offers insight into cross-frequency integration mechanisms in the brain. At 7 days post-lesion, we observed a significant increase in PAC, indicating changes in the correlation of high-frequency amplitudes by the phase of low-frequency oscillations following the lesion. After the focal ischemic lesion, slow oscillations appeared to “drive” faster activity (gamma/ripple oscillation) than under normal conditions. PAC is increasingly being studied as a biomarker of functional connectivity, as it reflects how different frequency bands interact and coordinate neural processes (Sotero, 2015; Amiri et al., 2016; Salimpour and Anderson, 2019). For example, strong delta–beta phase-amplitude coupling may reflect a mechanism by which slow cortical rhythms regulate the excitability of neuronal populations, synchronizing the timing of high-frequency bursts that are typically associated with active processing (Whittingstall and Logothetis, 2009; Palva and Palva, 2018). Recent evidence in humans confirms the relevance of such post-stroke phenomena: Mark et al. (2024) reported that in stroke patients, delta–beta PAC between the prefrontal cortex and motor cortex increased during the early stages of rehabilitation. Although this coupling tended to decline over the course of hospitalization, it remained elevated compared to healthy controls at discharge. We can speculate that enhanced cross-frequency coupling in our own data may also represent a beneficial compensatory mechanism or serve as an indicator of the integrity of residual circuits, even if this must be properly demonstrated through appropriate behavioral assessment. In line with this framework, it is plausible that the selective boost in S1 → RFA PAC is the outcome of the sensory cortex assuming a leading role in driving premotor activity after CFA output is lost. This would imply that, following damage to CFA, feed-forward information flow from sensory cortex toward motor areas assumes a predominant role to facilitate residual motor execution in the absence of primary motor output from CFA. Indeed, neurophysiological studies indicate that intact S1 and premotor areas can “stand in” for a lesioned CFA during motor recovery (Guggenmos et al., 2013; Hayley et al., 2023). Although our experiment involved only intrinsic plasticity, the heightened S1 → RFA drive we observed suggests that the brain is spontaneously reinforcing this pathway to possibly compensate for the loss of direct motor commands. Conversely, the relatively unchanged RFA → S1 coupling may indicate that feedback from premotor to sensory cortex remains largely stable, as sensory processing in S1 continues to be driven mainly by peripheral inputs and other intact networks. All our reported changes occur during SWS, a state critical for synaptic homeostasis and network reorganization.

When trying to link sleep patterns to brain activity, our analysis revealed that a focal CFA lesion disrupts the association between sleep duration and network centrality in a frequency- and region-specific manner. In S1, a strong inverse correlation between PLV-based centrality and SWS duration was present at baseline across all frequency bands, suggesting a tight coupling between local functional integration and sleep homeostasis in the somatosensory cortex. Following the lesion, this relationship was significantly weakened and remained disrupted through day 14, as confirmed by robust day-to-day contrasts across delta to gamma bands. Notably, in the gamma band, the lesion not only abolished the inverse relationship but appeared to invert it, reflecting a potentially maladaptive network reorganization of fast-frequency synchrony in relation to sleep drive. This suggests a sustained functional uncoupling of S1 from low frequency, global sleep-related dynamics, together with an impaired local regulation or loss of afferent input critically related to sleep. RFA, in contrast, showed weaker and frequency-limited positive associations at baseline, particularly in the beta and gamma ranges. These correlations were abolished after the lesion and also did not recover over time, indicating a flattening of functional coupling rather than a reorganization. The disruption in gamma in particular, though statistically significant, was less extensive than in S1, consistent with RFA’s relatively lower involvement in sleep-PLV centrality coordination. Another interpretation of these findings relates to the occurrence of plasticity-inducing events. In the baseline condition, animals were not exposed to novelty that could form new memory traces, whereas lesion animals experienced a sudden and drastic change in motor circuitry. The inverse correlation between PLV centrality (a proxy for a node’s network importance) and sleep duration at baseline may thus reflect synaptic homeostasis processes during sleep, particularly widespread synaptic downscaling (Ribeiro, 2012; Tononi and Cirelli, 2014). In contrast, the post-lesion reversal of this relationship could indicate that sleep promotes focalization of network activity in specific nodes (notably S1), thereby supporting plastic changes to compensate for the lost motor output. Together, these findings corroborate the notion that lesions alter not only local network architecture (Grefkes and Fink, 2014) but also its relevance in the context of sleep function (Khot and Morgenstern, 2019), in ways that are sustained, asymmetrical, and frequency specific.

Overall, the modifications we observed tended to be subtle, with some of our results not achieving statistical significance, but just trends. This could be attributed to the ET-1 model of ischemia used, which, despite being focal, often elicits modest behavioral impairments and may not produce extensive disruption of larger networks. This may also explain part of the variability observed in the results. Moreover, assessment of brain activity in additional time points would certainly contribute to further clarifying the temporal trajectory of network reorganization following stroke, and larger cohorts will be essential to validate these findings and link them to behavioral outcomes. On the other hand, our results provide a glimpse of the changes in the acute and subacute phases, the time periods that are often targeted by neuromodulation therapeutic approaches (Ward, 2017). On a correlated note, control animals should ideally be recorded also on additional time points after day 7. Although a 1-week recovery period is widely considered to be enough for the normalization/stabilization of important physiological variable after microelectrode implantation (Williams et al., 2007; Makowiecki et al., 2015), a direct observation of brain activity in these animals in a later stage would shed additional light into the issue, especially given conflicting evidence from human research (Cook et al., 2013).

To conclude, in this work, we employed a multifaceted electrophysiological approach that combines spectral power analysis, connectivity, and cross-frequency couplings with the goal of characterizing how brain networks reorganize during sleep after focal ischemia. We particularly focused on areas that, although taking part in impacted networks (the sensorimotor loop), were not directly targeted. Our results bear translational relevance, as they not only align with clinical data but also point toward possible directions for developing objective biomarkers of dysfunction and recovery (Silasi and Murphy, 2014). Moreover, our results open to further hypotheses regarding the role of sleep oscillations in supporting spontaneous post-stroke plasticity. Future work should test these hypotheses through longitudinal behavioral correlations and translational approaches, including the development of neurostimulation paradigms that exploit sleep-dependent network dynamics as potential therapeutic targets (Chiappalone et al., 2022; Carè et al., 2024).

## Data Availability

The raw data supporting the conclusions of this article will be made available by the authors, without undue reservation.

## Footnotes

^1^ https://github.com/barbaLab/nigeLab
