# Supplementary material for: Impact of ischemic lesion on sleep related connectivity in the sensorimotor cortex
Source: Front Neurosci. 2025 Nov 4;19:1661458. doi: 10.3389/fnins.2025.1661458 (PMC12623369; doi:10.3389/fnins.2025.1661458)
Supplement: Supplementary file 1 [file Data_Sheet_1.docx]

Supplementary Material

# Supplementary Figures and Tables

## Supplementary Figures

*
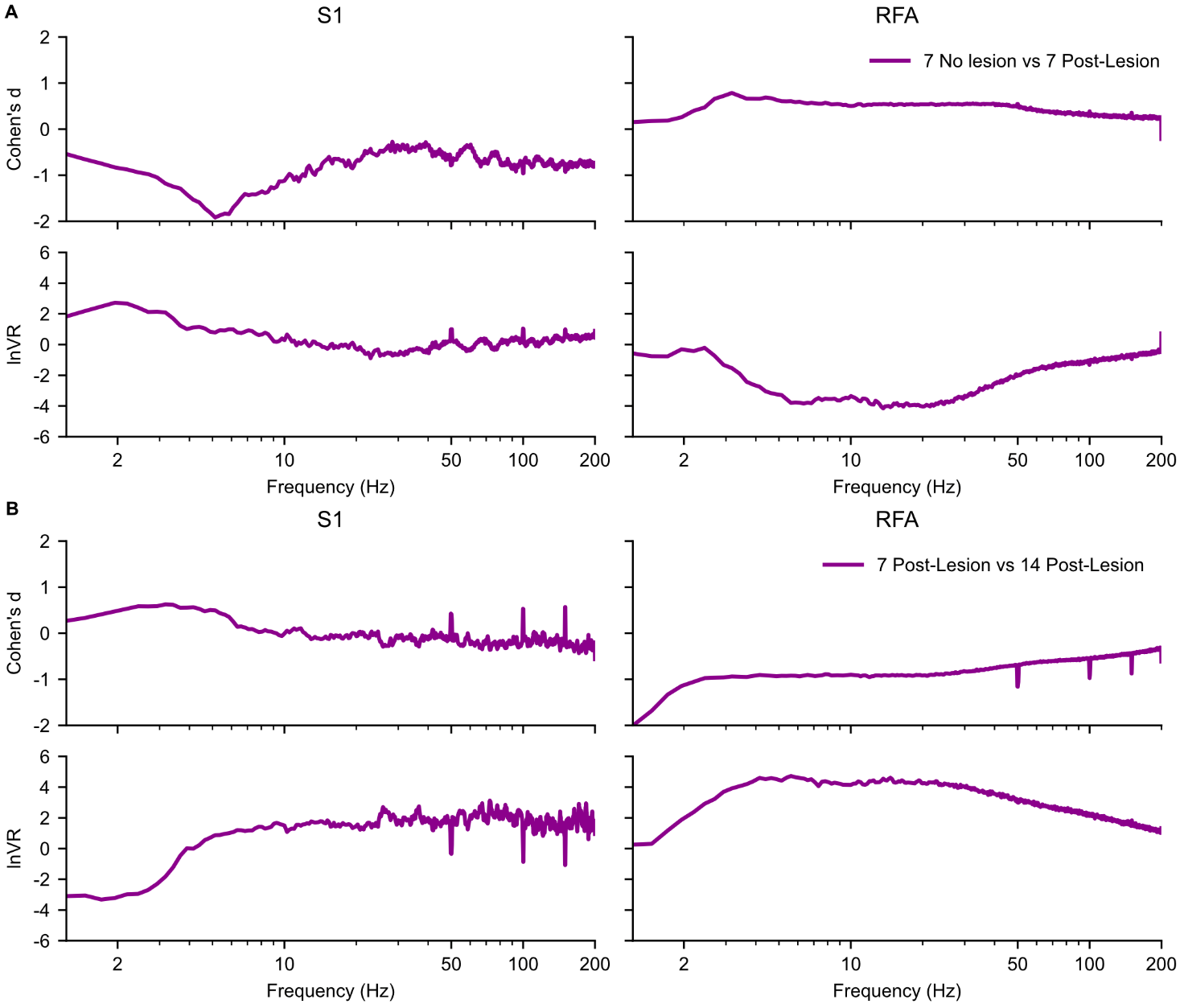
*

Supplementary 1 Support statistical analysis of PSD. Comparisons of power spectral density between no lesion vs 7 days post-lesion (panel A) and between 7 vs 14 days post-lesion (panel B). Each panel is organized in a 2×3 grid, with rows representing analysis type: (1^st^ row) Cohens’ d effect size (2^nd^ row) log-variance ratio (lnVR) and columns corresponding to brain regions and inter-areal connections. Columns represent S1 (left), RFA (right).

*
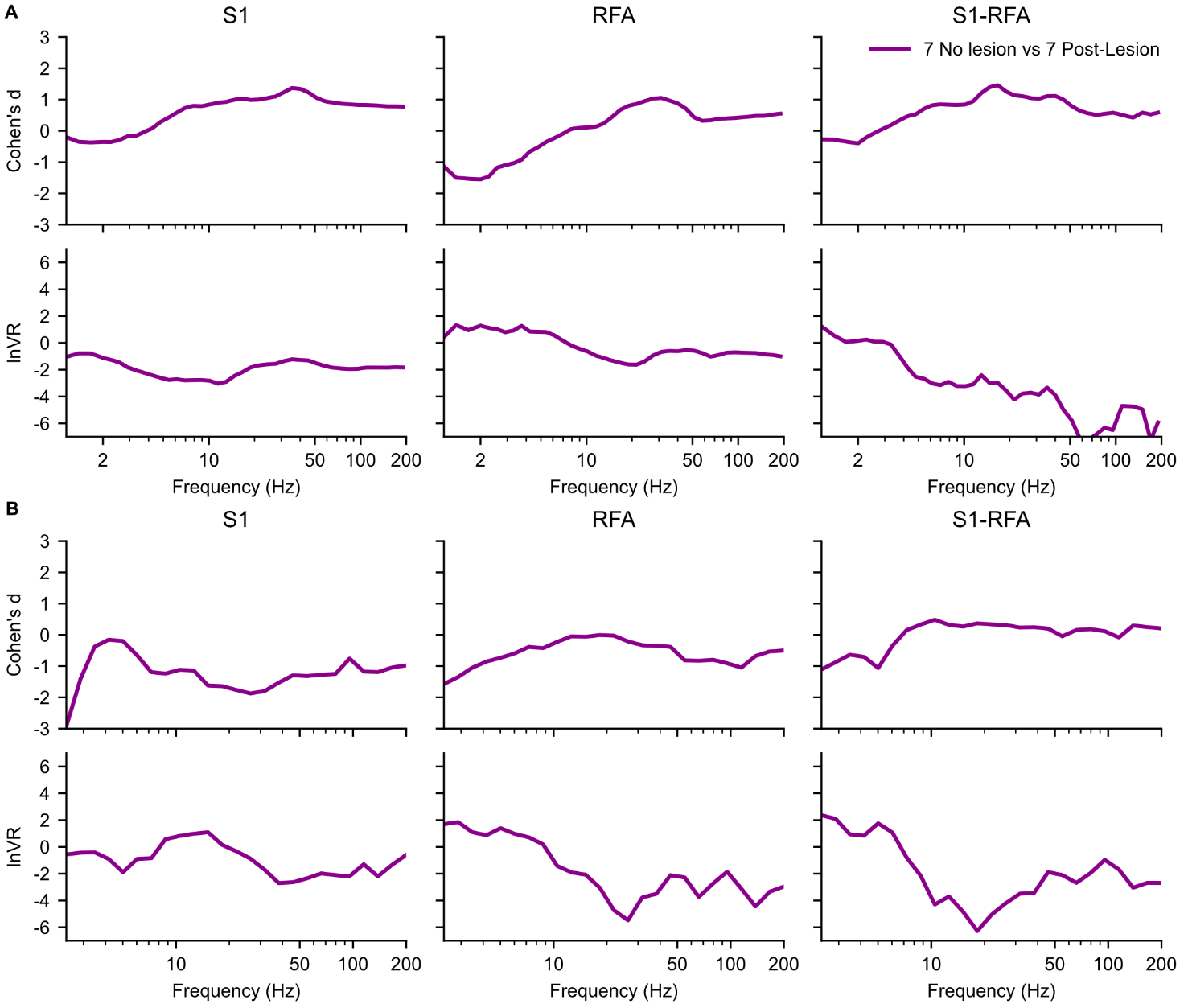
*

Supplementary 2 Support statistical analysis. Comparisons of phase-locking value (PLV; panel A) and phase–amplitude coupling (PAC; panel B) between no lesion and 7 days post-lesion. Each panel is organized in a 2×3 grid, with rows representing analysis type: (1^st^ row) Cohens’ d effect size (2^nd^ row) log-variance ratio (lnVR) and columns corresponding to brain regions and inter-areal connections. For PLV (A), columns represent S1 (left), RFA (center), and connectivity between the two regions (right). For PAC (B), columns represent S1 (left), RFA (center-left), directional coupling from S1 to RFA (center-right).


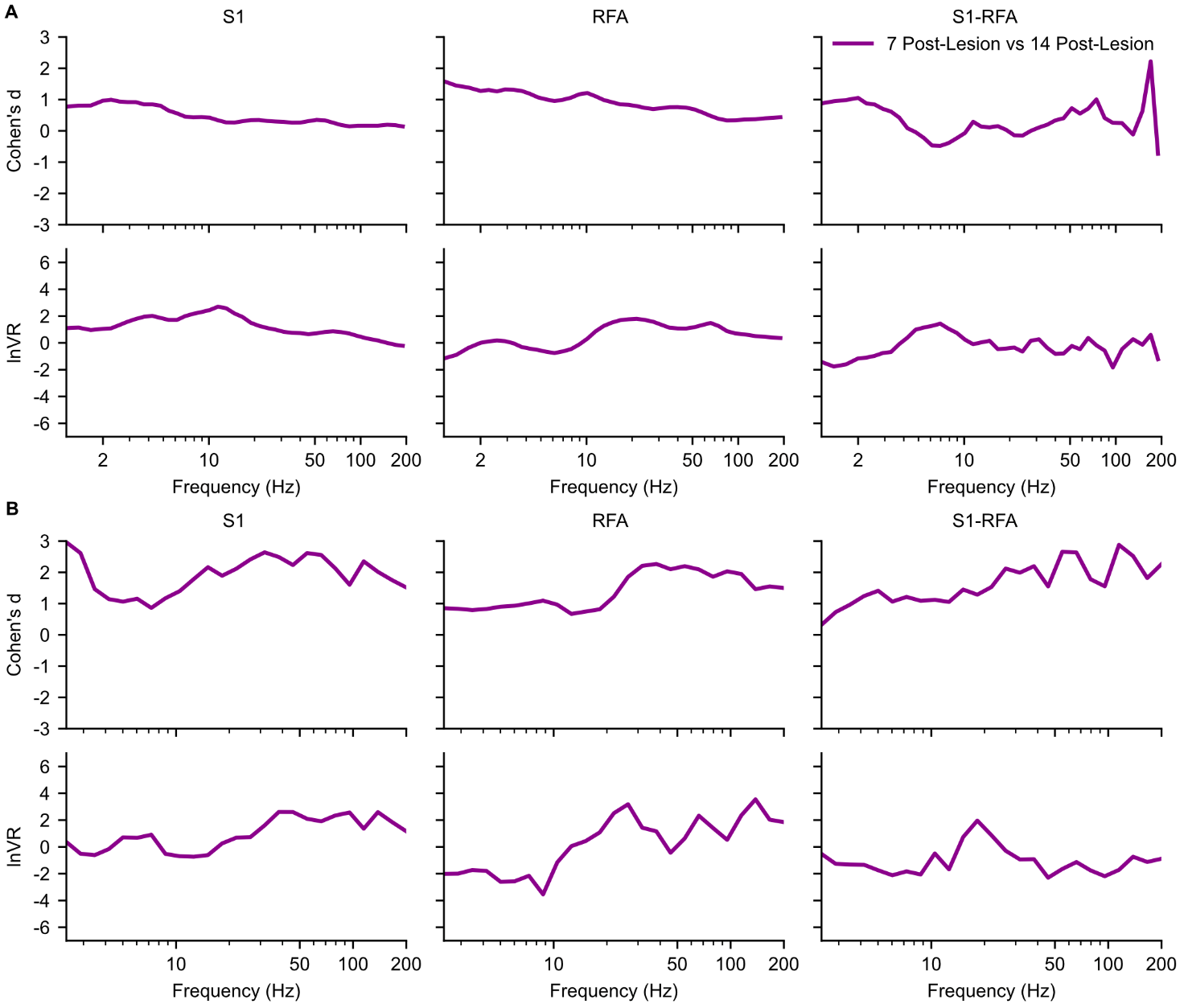


Supplementary 3 Support statistical analysis Comparisons of phase-locking value (PLV; panel A) and phase–amplitude coupling (PAC; panel B) between 7 vs 14 days post-lesion. Each panel is organized in a 2×3 grid, with rows representing analysis type: (1^st^ row) Cohens’ d effect size (2^nd^ row) log-variance ratio (lnVR) and columns corresponding to brain regions and inter-areal connections. For PLV (A), columns represent S1 (left), RFA (center), and connectivity between the two regions (right). For PAC (B), columns represent S1 (left), RFA (center-left), directional coupling from S1 to RFA (center-right).

*
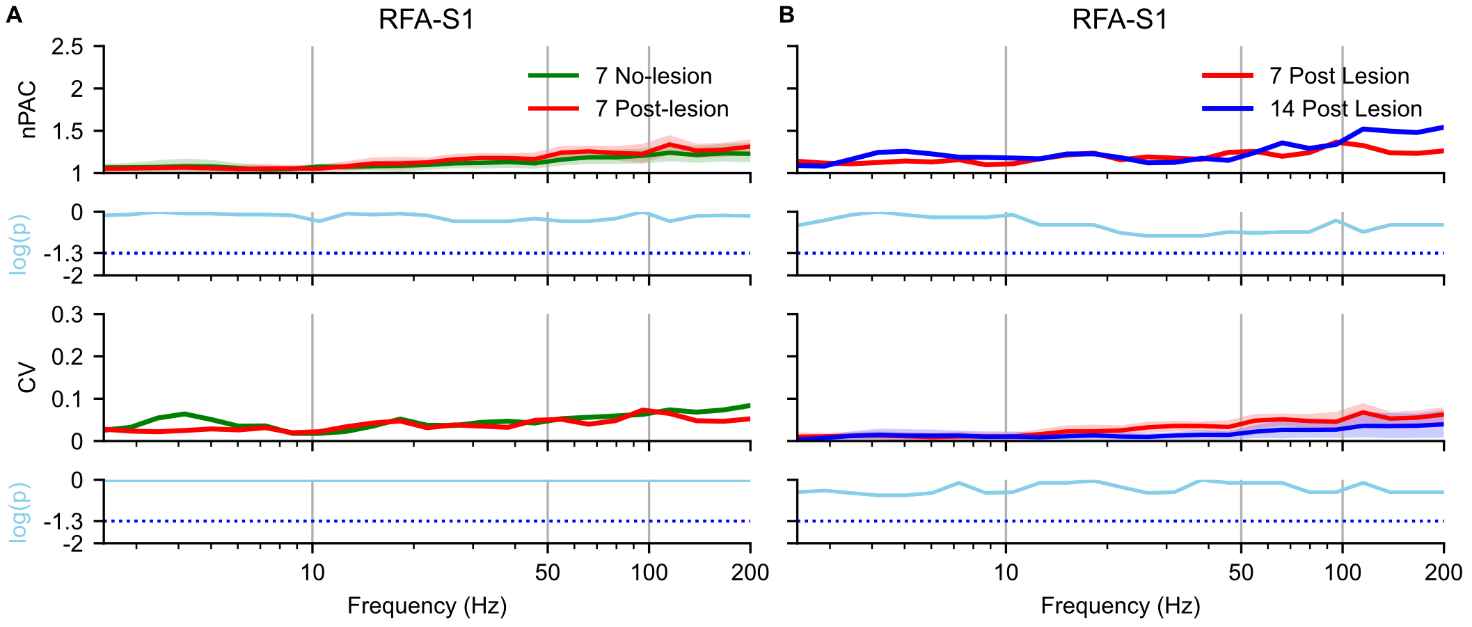
*

Supplementary 4. Spectral analyses and statistical comparisons of PAC between RFA and S1. Directional phase–amplitude coupling (PAC) between RFA and S1, across different post-lesion conditions: comparison between no lesion animals (green, n = 5) with animals recorded 7 days post-lesion (red, n = 4); Panel A, comparison between animals recorded 7 days post-lesion (red, n = 4) with those recorded 14 days post-lesion (blue, n = 4). Each panel is organized with 4 rows corresponding to different analytical measures. The first row shows the PAC spectral profile, where solid lines indicate group means and shaded areas represent 97% confidence intervals obtained via bootstrap resampling (n = 1000). The second row displays log-transformed p-values (cyan) resulting from statistical tests comparing PAC spectra between groups. Specifically, Mann–Whitney U tests were used in Panel A, and Wilcoxon rank-sum tests in Panel B; in both cases, p-values were corrected for multiple comparisons using the Benjamini–Hochberg procedure (α = 0.05), with the cyan dotted line indicating the 0.05 threshold. The third row shows the coefficient of variation (CV) across animals, reflecting inter-subject variability within each group. The fourth row presents log-transformed p-values from tests assessing group differences in variance: Fligner’s test for Panel A and a permutation test on the logarithmic differences in variance for Panel B, also corrected using the Benjamini-Hochberg method.

## Supplementary Tables

Supplementary Table 1 Post hoc analysis of linear mixed-effects models assessing phase-locking value (PLV) across experimental conditions (No-lesion, day 7 lesion, and day 14 lesion) and brain areas (RFA and S1), for each frequency band. For each pairwise contrast and frequency, the table reports the estimated difference in PLV (estimate), standard error (SE), degrees of freedom (df), t-ratio (t.ratio), and associated p-value (p.value). P-values have been adjusted using the Benjamini–Hochberg
